# Demonstrating the role of symbionts in mediating detoxification in herbivores

**DOI:** 10.1007/s13199-022-00863-y

**Published:** 2022-09-13

**Authors:** M. Denise Dearing, Martin Kaltenpoth, Jonathan Gershenzon

**Affiliations:** 1 School of Biological Sciences, University of Utah, Salt Lake City, UT 84112 USA; 2 Department of Biochemistry, Max Planck Institute for Chemical Ecology, Hans-Knöll-Str. 8, 07745 Jena, Germany; 3 Department of Insect Symbiosis, Max Planck Institute for Chemical Ecology, Hans-Knöll-Str.8, 07745 Jena, Germany

**Keywords:** Symbiont-mediated detoxification, Gut microbiome, Herbivores, Herbivory, Microbes, Plant secondary metabolites, Toxins

## Abstract

Plant toxins constitute an effective defense against herbivorous animals. However, many herbivores have evolved adaptations to cope with dietary toxins through detoxification, excretion, sequestration, target site insensitivity and/or via behavioral avoidance. While these adaptations are often directly encoded in herbivore genomes, evidence is accumulating that microbial symbionts can reduce the dose of plant toxins by metabolizing or sequestering them prior to absorption by the herbivore. Here, we describe a few well-studied examples to assess such symbiont-mediated detoxification and showcase different approaches that have been used for their analyses. These include: (i) a host phenotypic route in which the symbiotic association is manipulated to reveal host fitness costs upon toxin exposure in the presence/absence of detoxifying symbionts, including function restoration after symbiont re-infection, (ii) a molecular microbiological approach that focuses on the identification and characterization of microbial genes involved in plant toxin metabolism, and (iii) an analytical chemical route that aims to characterize the conversion of the toxin to less harmful metabolites in vivo and link conversion to the activities of a detoxifying symbiont. The advantages and challenges of each approach are discussed, and it is argued that a multi-pronged strategy combining phenotypic, molecular, and chemical evidence is needed to unambiguously demonstrate microbial contributions to plant toxin reduction and the importance of these processes for host fitness. Given the interdisciplinary nature of the topic, we aim to provide a guideline to researchers interested in symbiont-mediated detoxification and hope to encourage future studies that contribute to a more comprehensive and mechanistic understanding of detoxification in herbivores and their symbionts.

“Our imagination is struck only by what is great but the lover of natural philosophy should reflect equally on little things” Alexander von Humboldt.

Plant based diets, while celebrated for their health benefits to humans, pose a considerable nutritional challenge to many herbivorous metazoans (Karasov and Martínez del Rio 2007). Notably, the herbivorous lifestyle of animals is enabled through symbioses with a wide variety, and typically a tremendous abundance of microbial partners, e.g., the gut microbiome (Karasov and Douglas 2013). For many herbivores, the majority of the energy present in plant material is inaccessible to their own enzymatic machinery and can only be utilized after microbial biotransformations of complex polysaccharides (cellulose, hemicelluloses, pectin, collectively known as fiber) into compounds that can be absorbed and further metabolized by the animal host (Demment and Van Soest 1985). Furthermore, the production of secondary compounds (metabolites) by plants can present even greater dietary challenges when these molecules occur at levels that negatively impact herbivore physiology, reproduction and ultimately fitness (Wink 2008). While the importance of microbes in metabolizing polysaccharides has been known for over a hundred years, and has been documented across numerous taxa from arthropods to vertebrates, comparable demonstrations of microbial degradation of plant toxins are still scarce (Itoh et al. 2018b; Dearing and Weinstein 2022).

The goal of this *Perspectives and Ideas* paper is to provide a foundation for those interested in investigating the role of detoxification pathways in microbial symbionts that facilitate herbivory. Specifically, we explore whether microbial metabolism of dietary plant derived toxins could enable herbivory by reducing/eliminating the dose of toxins experienced by an herbivore. We approach this manuscript from different perspectives united by a common theme. Gershenzon’s work has focused on identifying the mechanisms employed by arthropod herbivores to circumvent plant toxins, such as detoxification, target-site insensitivity, excretion and specialized feeding behavior. Dearing’s early studies centered on how vertebrate herbivores either behaviorally manipulated or physiologically metabolized dietary toxins, with the latter leveraging standard pharmacological techniques. Kaltenpoth characterizes the interactions between symbionts and their arthropod hosts with respect to niche expansion, food exploitation and defense. Currently, the three of us share a common interest in understanding the role of microbes in metabolizing the dietary toxins ingested by their hosts, mostly herbivores (Berasategui et al. 2016, 2017; Kohl and Dearing 2016; Zhao et al. 2019; Weinstein et al. 2021; Stapleton et al. 2022). A brief historical summary on bacterial symbiont mediated detoxification is presented, followed by a few select examples in vertebrate and invertebrate hosts, and a set of criteria are defined to help researchers to firmly establish causality of the symbiont in contributing to detoxification that benefits the host. More comprehensive reviews of reported examples have been recently presented (Itoh et al. 2018b; Dearing and Weinstein 2022). While this manuscript focuses on bacterial symbionts, the concepts presented apply to fungal symbionts as well.

Plants attempt to defend themselves by producing compounds toxic to potential herbivores, and the idea that microbes associated with herbivores may enhance the detoxification capacities of their hosts is a long-standing one. Microbes are well known to metabolize otherwise indigestible material, such as polysaccharides in animal rumens, but much less attention has been devoted to their detoxification abilities. Over 50 years ago, ecologists William Freeland and Daniel Janzen proposed that mammalian herbivores in nature should host microbial symbionts with detoxification capabilities to enable the ingestion of toxic plants (Freeland and Janzen 1974). Somewhat simultaneously, Christine Janis suggested that the detoxification of plant forage by bacteria may have been the key selective force driving the origin of the foregut in ruminants (Janis 1976). This hypothesis was based on the high toxin loads predicted to be present in the diets of proto-ruminants, and thus detoxification was expected to be more important than other bacterial functions such as nitrogen recycling. Meanwhile, animal scientists were characterizing the community structure and function of microbes in the rumen of domesticated herbivores mostly with an emphasis on fiber fermentation, while a handful of other researchers were beginning to explore detoxification of plant toxins by rumen microbes (Jones and Megarrity 1983; Van Soest 1994). In parallel, the field of insect symbiosis had been developing over the past 100 years, where researchers were keenly aware of the taxonomic distribution as well as the various localizations and transmission routes of microbial symbionts (Buchner 1965). However, knowledge on the functional roles that microbes played in symbiotic interactions at that time remained mostly limited to nutrient supplementation and cellulose digestion (Buchner 1965).

We are now beginning to be able to interrogate and understand “detoxifying” symbioses in herbivores. Many species of herbivores show promise for the possibility of hosting symbionts that could enhance detoxification (Itoh et al. 2018b). Moreover, we have the tools and expertise to identify, characterize, and manipulate the components of these symbioses (Elston et al. 2022; Stapleton et al. 2022). Before exploring examples of these prospective symbioses, we provide broad definitions of key terms used in this discussion with the recognition that these simple descriptions cannot address the complexity found in nature and that other disciplines may use these terms differently (Box 1).

**Table:** 

**Box 1**. Definitions of key terms	
Plant Toxins: Plant chemicals poisonous to herbivores and also other organisms such as symbiotic microbes, via a variety of mechanisms. As most toxins are dose dependent, it is necessary to establish whether native concentrations of toxins are dangerous to the herbivore under study in order to know whether detoxification benefits the herbivore or not. Detoxification: Chemical modification of a toxin that makes it physiologically less harmful. This process can be executed by the herbivore host, the microbial symbiont, or both. Symbiosis: Individuals of different species living together in a reasonably close association for an extended period of time. “Detoxifying symbiosis”: Symbiont-mediated modification of a toxin that benefits the host. Microbial symbionts can be present in or on the host or in the host’s environment.

“We are not me-

We are we.” (Gorman 2021)

Microbes that degrade toxins ingested by herbivores are likely to be widespread among herbivorous taxa because microbes with the requisite metabolic abilities may frequently be present in nature (Hammer and Bowers 2015). Local environments of high toxin abundance, e.g., in the vicinity of toxin-producing plants, should exert strong selection pressure on microbes for degradation capability (Hansen and Enders 2022). Moreover, the ability for detoxification should spread rapidly within microbial communities due to short generation times and horizontal gene transfer. Microbes also possess the advantage of catalyzing many types of reactions that are not yet known in vertebrate and invertebrate herbivores, such as isothiocyanate hydrolases (glucosinolate detoxification, Welte et al. 2016), caffeine demethylases, (caffeine detoxification, Ceja-Navarro et al. 2015), and catechol dioxygenases (flavonoid detoxification, Wadke et al. 2016). In addition, microbes produce laccases that can perform phenolic detoxification (De Fine Licht et al. 2013); these enzymes are not present in vertebrate herbivores. Such microbial processing of toxins may be more resource and energy-efficient than direct metabolism by herbivores. For example, most herbivores detoxify the biologically active hydrolysis products of glucosinolates, the isothiocyanates, by formation of glutathione conjugates, which are then metabolized and excreted such that one mole of cysteine is lost for each mole of isothiocyanate detoxified (Jeschke et al. 2016, 2017). As herbivores are often nitrogen limited, the loss of an amino acid, i.e., cysteine, during the process of detoxification represents a significant cost (Jeschke et al. 2016). By contrast, microbial detoxification converts isothiocyanates in a one-step reaction to non-toxic amines, which can then be excreted or further metabolized without expenditure of cysteine (Jeschke et al. 2016; Shukla and Beran 2020).

## Select examples of symbiont-mediated detoxification

One of the clearest examples of symbiont mediated-detoxification is the degradation of oxalate by gut microbes (Allison et al. 1985). The vast majority of herbivore hosts do not produce enzymes that can modify this compound. Oxalate, a simple acid produced by some plant species, causes physiological and structural damage to animal cells and tissues when ingested (Franceschi and Nakata 2005). Indeed, the calcium oxalate common in dietary items of humans such as spinach or tea, is the leading precursor of kidney stones (Moe 2006). Oxalate degradation has been documented in over twenty different microbes, along with many of the genes and enzymes responsible for oxalate metabolism (Miller and Dearing 2013). The first discovered gut microbe with this property was *Oxalobacter formigenes*, isolated from the gut communities of several mammals including humans (Allison et al. 1985). Since then several other gut microbes from herbivores have been documented to have oxalate degrading capabilities (Miller and Dearing 2013). For example, among herbivorous insects, the extracellular gut symbiont *Ishikawaella capsulata* of plataspid stinkbugs has an oxalate decarboxylase (Nikoh et al. 2011). Several *Lactobacillus* species capable of degrading oxalate have been isolated from herbivorous woodrats that consume high levels of oxalate in diets of cactus, and the abundances of these microbes are governed by levels of dietary oxalate (Miller et al. 2014, 2016). Given that dietary oxalate is the leading cause of kidney stones in humans, and that *O. formigenes* is disappearing in many human populations likely to antibiotic use (PeBenito et al. 2019), bioprospecting in the microbial communities of herbivores might result in the discovery of novel microbes that could be leveraged to improve human health.

Another intriguing example of symbiont-mediated detoxification is that of caffeine detoxification by the coffee berry borer. The purine alkaloid 1,3,7-trimethylxanthine, better known as caffeine, is produced by plants across 13 different orders (Ashihara and Suzuki 2004), including the commercially important coffee (*Coffea arabica* and *C. canephora*), tea (*Camellia sinensis*), and cocoa plants (*Theobroma cacao*). In nature, caffeine likely serves as an anti-herbivory defense molecule, given its deterrent and toxic effects on arthropods. While many insect species occasionally feed on parts of coffee plants, the coffee berry borer (*Hypothenemus hampei*, Curculionidae) is the only known species to complete its entire life cycle solely on coffee beans, apparently thriving on the high concentrations of caffeine in their diet. In an elegant study, Ceja-Navarro et al. (2015) investigated the involvement of gut microbial symbionts in this beetle’s ability to degrade caffeine, providing one of the most convincing cases of symbiont-mediated detoxification of natural products in insects to date. After delivery of caffeine in an artificial diet to laboratory-reared individuals, the beetles’ frass lacked detectable levels of caffeine, indicating that it was metabolized rather than excreted or sequestered. Removal of the gut microbiota by antibiotic treatment abolished caffeine degradation and resulted in severely reduced fitness of the beetles (Ceja-Navarro et al. 2015). Cultivation of gut bacteria from the beetles yielded a *Pseudomonas fulva* strain that was able to utilize caffeine as its sole carbon and nitrogen source. Importantly, re-infection of *P. fulva* into the beetles restored caffeine break-down but not the beetle’s fitness, indicating that either the antibiotic treatment had direct adverse effects on the beetle or that other gut microbes fulfil important additional functions that could not be compensated by *P. fulva* alone (Ceja-Navarro et al. 2015). Molecular analyses revealed a caffeine demethylase gene *ndmA* in *P. fulva* expressed *in beetlo*, providing a promising candidate for the conversion of caffeine to theobromine - the first step in caffeine conversion to xanthine. Subsequent studies found *Pseudomonas* strains to be variably abundant across coffee borer populations (Marino et al. 2018; Vega et al. 2021; Mejia-Alvarado et al. 2021), and additional caffeine-degrading bacteria were isolated from the beetles (Vega et al. 2021). This finding supports the notion that detoxifying symbionts may be dynamically recruited from the host plant, where the toxin exerts selection on microbes for degradation capability. Interestingly, some of the microbial isolates lacked caffeine demethylase genes and thus may metabolize caffeine via other biochemical pathways (Vega et al. 2021). While these studies clearly document that microbes can degrade dietary toxins ingested by the beetle, the fitness contributions any of these putative detoxifying symbionts to the beetle remain to be elucidated.

The most celebrated example of symbiont-mediated detoxification among vertebrate herbivores is that of the bacterium *Synergistes jonesii* and the degradation of mimosine and its toxic dihydroxypyridine (DHP) derivatives. Mimosine is produced in high quantities by leucaena, (*Leucaena leucocephala)*, a fast growing shrub commonly used as forage in tropical areas despite its strong antimitotic effect on cell division in mammals (McSweeney et al. 2002). *S. jonesii* was initially isolated and identified from rumens of Hawaiian goats fed diets of leucaena. Over 50 years ago, animal scientists initiated a research program (that continues to this day), to improve outcomes for livestock fed leucaena through symbiont-mediated detoxification. This effort was based on preliminary observations of differential tolerances to mimosine poisoning among ruminants from different regions (reviewed in Shelton et al. 2019). Transfers of rumen fluid from resistant ruminants to sensitive ones appeared to improve resistance to mimosine poisoning; however, it is worth noting that because of funding limitations, the sample sizes in the experimental treatments were limited to one individual from each of two sensitive domestic species, with no controls (Jones and Megarrity 1986). In subsequent studies, *S. jonesii* was isolated from the rumen of resistant goats, and cultures of this microbe were documented to degrade toxic derivatives of mimosine (Allison et al. 1990, 1992). Based largely on these results, researchers concluded that the resistance observed in Hawaiian goats was due to symbiont-mediated detoxification by *S. jonesii*, and a large scale program was established in Australia to inoculate domestic animals with a commercial probiotic culture derived from the rumen fluid of cattle that had been inoculated with *S. jonesii* (Klieve et al. 2002). However, as several additional microbes that degrade DHP have since been identified from these rumen communities, the contribution of *S. jonesii* to the overall symbiont-mediated detoxification of mimosine remains to be clearly quantified (Dominguez-Bello and Stewart 1990; Derakhshani et al. 2016; Aung 2019). Furthermore, recent work on the role of the herbivore with respect to the direct detoxification of mimosine suggests that microbially mediated detoxification of mimosine, particularly by *S. jonesii*, may not be as extensive, or even required, as was originally thought (Shelton et al. 2019). The role of symbiont-mediated detoxification with respect to mimosine highlights the difficulties in independently quantifying the contributions of the symbiont and the host particularly in large animals where sufficient replication of experimental subjects can be especially costly. And it also underscores the importance of including host metabolism in these studies. These examples represent some of the most well-known cases of symbiont mediated detoxification in herbivorous mammals and insects, while highlighting some of the challenges in unambiguously documenting the role of the symbiont in detoxification.

## What’s needed to demonstrate detoxifying symbioses

“Everything is hard before it is easy” Johann Wolfgang von Goethe.

While there are many promising examples of microbially mediated detoxification, firmly establishing such symbioses, requires a substantial effort consisting of a wide portfolio of techniques and expertise, i.e., a scientific symbiosis. Ideally, documenting symbiont-mediated detoxification would follow the general logic of Koch’s postulates modified for determining causation in human microbiome studies (Neville et al. 2018). This includes.

1. Identification of the gene(s) in symbiont(s) responsible for detoxification coupled with identification of the dietary compounds on which the symbiont acts.
2. Removal of the gene/symbiont of interest, followed by the introduction of a genetically modified gene/symbiont that can carry out the detoxification reaction into the host, and then measuring the physiological consequences on the host without and with the modified gene/symbiont

Below we outline the various approaches that have been taken, and the advantages and limitations of each (summarized in Box 2). We end with a set of criteria that we feel necessary to clearly document symbiont-mediated detoxification.

## Three routes for investigations of symbiont-mediated detoxification

Generally, researchers have employed one or two of the three different approaches described below to address symbiont-mediated detoxification in animals, with the choice usually based on the available expertise and equipment, as well as the properties of the study system. All three approaches require that the negative impact of the plant toxin of interest be demonstrated in ecologically relevant concentrations. While this may seem trivial, it can in practice present considerable challenges. After all, if a toxin is indeed detoxified by the host or its symbiont, the host is likely to suffer little or no deleterious effects, so the toxicity will go unnoticed. Thus, to demonstrate that a plant metabolite is indeed a toxin, its activity should be assessed either in bioassays with non-specialist herbivores closely related to the animal of interest, or in conjunction with targeted manipulation of the mechanism for detoxification in the target species. Once the toxin is identified, symbiont-mediated detoxification can be evaluated from the phenotypic, the molecular/microbiological, and/or the chemical side (Box 2).

**Table:** 

**Box 2**. Routes for investigating symbiont-mediated detoxification		
Host Phenotype	Microbial/Molecular	Chemical
• characterize microbial community of host	• characterize microbial community of host	• quantify toxin and potential metabolites
• manipulate presence of symbionts, e.g. with antibiotics or by manipulating transmission	• identify putative symbionts of host	• administer isotopically-labeled toxin
• assess performance of symbiotic and aposymbiotic hosts in presence/absence of toxin	• determine and manipulate symbiont genes involved in detox pathways	• link detox reaction to symbiont (by molecular or biochemical evidence or via imaging)
→ demonstration that symbiont depleted hosts have lower fitness when toxin is present	→ elucidation of detoxification pathway (useful to manipulate the detox process)	→ establish precise chemical fate of toxin; metabolites can be tested for toxicity

### The host phenotypic route

This approach relies on the manipulation of the symbiosis and the assessment of its effects in the presence and absence of the toxin, respectively. It usually starts with a culture-dependent and/or culture-independent characterization of the microbial community associated with the host, followed by manipulation via antibiotic treatment or disruption of the natural route of vertical or horizontal symbiont transmission or acquisition from the environment (Dowd and Shen 1990; Kohl et al. 2014; Berasategui et al. 2016; Shukla and Beran 2020). If symbiont-mediated detoxification occurs, symbiont-depleted hosts should suffer fitness costs in the presence, but less so in the absence of the toxin. Importantly, however, proper controls are essential to exclude non-target side effects of the symbiont elimination procedure (Shukla and Beran 2020). Suitable approaches include the reconstitution of the natural microbial community (e.g., via fecal transplants, Kohl et al. 2014) and subsequent monitoring, or the targeted re-infection with a single cultured microbe that is the candidate symbiont for detoxification (Ceja-Navarro et al. 2015).

The phenotypic route has two advantages over the other approaches, making it a widely used strategy for assessing symbiont-mediated detoxification: (i) it is technically less demanding than the other two approaches, and (ii) it yields important information on the fitness consequences of detoxification, and thereby, provides insights into the ecological and evolutionary implications of the symbiosis. On the downside, however, detoxification is difficult to demonstrate via the phenotypic route if symbionts confer additional benefits, and it is impossible to distinguish direct and indirect effects, i.e., to assess whether the microbes really detoxify or enable the host to cope with the toxin by alternative mechanisms. Furthermore, this approach requires that (i) the animal host can be reared under controlled conditions, (ii) the toxin is available for experimentation in sufficient quantities (either commercially available or extracted from the plant), (iii) a toxin-free diet either artificial or natural for the animal is available, (iv) the toxin can be delivered to the animal in the diet, (v) the relevant microbial taxa can be removed from the animal, and (vi) re-infection of the animal with the relevant microbes is possible (either from cultivated individual symbionts or a natural community).

### The molecular/microbiological route

The second approach relies on molecular methods to identify and manipulate detoxification genes in the symbiont. It starts with a thorough taxonomic and functional characterization of the microbial community associated with the host animal by cultivation-based approaches in combination with full-genome sequencing of the isolates or meta-genomics or meta-transcriptomics of the microbial community. Identification of putative detoxifying symbionts can be accelerated by using selective toxin-containing media for microbial isolations (Ceja-Navarro et al. 2015; Kohl et al. 2016; Vega et al. 2021). If the media contains the toxin as the sole carbon and/or nitrogen source, only microbial taxa that can metabolize the toxin are able to grow, obviating the need for phenotypic assays later on. To identify the genes underlying detoxification, the symbiont genome can be screened for known pathways involved in the break-down of toxins (Adams et al. 2013), or the expression of genes can be assessed in the presence and absence of the toxin, respectively. Alternatively, genome-wide manipulation (e.g. via transposon-insertion sequencing) can be leveraged to identify candidate genes (Ganesan et al. 2021). Following identification, targeted manipulation of candidate genes and complementation of knock-out mutants allows for the determination of biochemical pathways underlying genetic basis of detoxification (Sato et al. 2021).

While failing to provide information on the occurrence of symbiont-mediated detoxification in vivo or its relevance for host fitness, the advantage of the microbiological/molecular approach lies in the elucidation of the underlying molecular pathways. However, it relies on (i) the culturability of the symbiont in vitro, (ii) the ability to identify candidate detoxification genes, and, crucially, (iii) the genetic tractability of the symbiont. In reality, most reported studies using this approach end after isolation of the microbes and demonstration of their detoxifying ability in vitro, with further genetic manipulation and characterization rarely included.

### The chemical route

The third route takes a chemical approach, i.e., characterization of the conversion of the toxin to less harmful metabolite(s) in vivo. Classically, this can be achieved by following isotopically-labeled precursors or quantifying known catabolites in well-characterized detoxification pathways by mass spectrometry (Shukla and Beran 2020). However, subsequently, toxin catabolism needs to be linked to a symbiont based on (i) molecular evidence, i.e., symbiont genes are characterized that encode detoxifying enzymes, and/or the likely catabolic pathways are not encoded in animal genomes; (ii) biochemical evidence, i.e., a protein extract from microbial culture can catalyze the detoxification reaction; and/or (iii) chemical localization, i.e., tracing a labeled toxin and its break-down products in vivo, and demonstrating localization within symbiont cells by autoradiography, mass-spectrometry imaging, fluorescence or Raman microscopy, or alternative localization techniques.

Technically demanding, this approach has been more rarely (as compared to the two other approaches mentioned) used in order to establish symbiont-mediated detoxification, although its virtue in unambiguously establishing toxin catabolism and ability to follow toxin break-down products is undeniable. A major downside of this approach are difficulties in obtaining labeled toxin or its precursors required for assessing the chemical conversions. Furthermore, linking toxin break-down to a particular member within the host-microbiome can be extremely difficult without experimental manipulation of the community, especially given the challenges in localizing toxin break-down products in situ.

Most existing studies on symbiont-mediated detoxification use one of these approaches or parts thereof. Ideally, however, a multi-level approach should be used toward unravelling symbiont-mediated detoxification that combines aspects of all three approaches. In particular, research should include a thorough phenotypic characterization of the benefits provided by the symbiont to the host by quantifying proxies of fitness such as growth rate, developmental time, body mass, survival, or reproductive output. In addition, molecular and/or chemical evidence for the symbiont-mediated detoxification pathway should be provided. Together, such results would provide causal evidence for symbiont mediated detoxification. However, as each of the three approaches presents different challenges and comes with different requirements for the study system, a comprehensive characterization and unambiguous demonstration of symbiont-mediated detoxification has rarely been achieved.

### Case study

Notably, however, the break-down of the synthetic insecticide fenitrothion by the concerted action of the bean bug and its *Burkholderia* gut symbiont sets a high standard for establishing symbiont-mediated detoxification that has yet to be realized for the symbiotic break-down of a natural plant toxin (Kikuchi et al. 2012; Sato et al. 2021). In this symbiosis, the bacterial partner is acquired from the environment every generation (Kikuchi et al. 2007), providing excellent opportunities for experimental manipulation. Infection of the bugs with fenitrothion-degrading *Burkholderia* results in immediate fitness benefits to the host when confronted with the insecticide (Kikuchi et al. 2012), and bug populations were found to harbor resistant bacteria in regions with repeated fenitrothion exposure (Kikuchi et al. 2012; Itoh et al. 2018a). Importantly, cultivation, genome sequencing, and genetic manipulation of the symbionts allowed for identification of the fenitrothion degradation pathway, and bacterial knock-out mutants failed to confer fenitrothion resistance to their host despite successful bacterial colonization of the mid-gut crypts (Sato et al. 2021). These experiments provided unambiguous evidence for symbiont-mediated detoxification in vivo and its impact on host fitness. Interestingly, in vitro and in vivo bioassays further revealed that the symbionts convert the insecticide fenitrothion into the bactericidal compound 3-methyl-4-nitrophenol, which is, in turn, excreted by the host, protecting the symbiont from intoxication by its own catabolite (Sato et al. 2021). Thus, the detoxification process includes contributions from the microbe that benefits the host, but also from the host that assists in the detoxification process.

This example illustrates another key point with respect to symbiont-mediated detoxification in herbivores, i.e., the acquisition of microbes from the environment. Many studies focus on identifying microbes found either in or on the herbivore; however, the plant tissues fed on by herbivores may be a good source of microbes capable of metabolizing the major toxins present in these plants (Mason et al. 2014; Hansen and Enders 2022). For example, a lepidopteran herbivore (*Lymantria dispar*) of aspen (*Populus tremuloides*) acquires bacteria associated with aspen foliage that are able to degrade the phenolic toxins of aspen leaves and thus improve herbivore performance (Mason et al. 2014, 2016). Such loose, horizontally-transmitted and acquired associations give herbivores flexibility in altering their complement of microbial symbionts depending on their diet (Mason et al. 2019) and may enable individuals to switch diets quickly.

In summary, this is an exciting time for exploring symbiont-mediated detoxification in herbivores. Many promising natural systems have been identified, and current tools and technology including metagenomics and microscopy, coupled with culture-based approaches allow for rigorous investigation. However, demonstration of symbiont-mediated symbiosis remains challenging and requires a range of tools and skills. A clear demonstration of these interactions will likely need to draw on a diversity of approaches and expertise. Expanding on the knowledge of symbiont-mediated detoxification in the future will undoubtedly advance our understanding of microbial symbionts as important sources of ecological innovation and shed light on their role in enabling herbivory across vertebrate and invertebrate hosts.
